# Les traumatismes fermés du rein: à propos de 55 observations

**DOI:** 10.11604/pamj.2014.17.127.3765

**Published:** 2014-02-22

**Authors:** Khalid Ksiri, Issam Goultein, Rachid Aboutaeib, Mohamed Dakir, Redouaune Rabii, Adil Debbagh, Saad Bennani, Fathi Meziane

**Affiliations:** 1 Service d'Urologie, CHU IBN Rochd, Casablanca, Maroc

**Keywords:** Rein, traumatismes, traitement conservateur, kidney, trauma, Conservative treatment

## Abstract

Le rein est l'organe le plus fréquemment atteint lors des traumatismes urologiques. Actuellement la prise en charge des traumatismes rénaux est de plus en plus conservatrice. Nous avons réalisé une étude rétrospective portant sur 55 cas de traumatisme fermé du rein, colligés au service d'urologie au CHU Ibn Rochd de Casablanca sur une période de 6 ans étalée entre 2006 et 2012. Le but de ce travail est d'analyser notre prise en charge thérapeutique et les résultats obtenus en les comparants à différentes séries de la littérature. Le but final étant d'améliorer la prise en charge des traumatismes fermés du rein au sein de notre établissement sanitaire. L’âge moyen était de 26 ans. Le sexe ratio (H/F) était de 10. Les étiologies étaient dominées par les accidents de la voie publique (27 cas), et les chutes (11cas). Cinq patients se sont présentés en état de choc et opérés en urgence. Tous nos patients ont eu une tomodensitométrie rénale permettant de classer les traumatismes selon l'AAST (American Association for the Surgery of Trauma): 25% de lésions grade I, 9% de lésions grade II, 38,6% de lésions grade III, 22,7% de lésions grade IV, et 4,5% de lésions grade V. Le traitement conservateur était efficace chez 40 cas, dont 6 ont bénéficié d'une montée de sonde JJ. Le traitement chirurgical était nécessaire chez 15 cas, 5 en urgence immédiate et 10 en urgences différées après une attitude conservatrice initiale. Après un recul moyen de 11 mois les patients traités de façon conservatrice ont évolué favorablement. Devant l’évolution favorable de nos patients ayant eu une simple surveillance et tenant compte des résultats de la littérature, nous pensons qu'en l'absence de lésions intra-abdominales associées ou d'instabilité hémodynamique, la simple surveillance reste la méthode thérapeutique de choix.

## Introduction

Les traumatismes sont les principales causes de mortalité chez le sujet jeune entre 1 et 44 ans. En urologie, le rein est l'organe le plus fréquemment atteint [1]. Le scanner spiralé avec injection de produit de contraste est le meilleur examen pour confirmer le diagnostic et apprécier l’étendue des lésions [2]. La classification utilisée pour décrire les traumatismes du rein est la classification de l'ASST (comité américain de chirurgie traumatologique) [3]. Le traitement des traumatismes fermés rénaux majeurs reste un sujet de débat: opérer ou surveiller. A travers une étude rétrospective concernant 55 cas de traumatismes rénaux fermés et à travers une revue de la littérature, nous étudierons les aspects diagnostiques et thérapeutiques récents des traumatismes fermés du rein.

## Méthodes

Entre janvier 2006 et octobre 2012, 55 patients ayant des traumatismes rénaux fermés de gravité variable et pris en charge dans notre formation, ont été inclus dans une étude rétrospective. Différents éléments ont été étudiés à savoir l’âge, e sexe, le côté lésé, l’étiologie, la présence de lésions associées (viscérales, osseuses), les signes cliniques, le bilan radiologique permettant de définir le grade ésionnel et enfin le traitement utilisé ainsi que les omplications. Après leur sortie, les patients ont été suivis régulièrement en consultation avec un examen clinique, un dosage de la créatinine sérique, et un examen tomodensitométrique (TDM), une échographie, ou parfois une scintigraphie.

## Résultats

Dans notre série, le sexe masculin était prédominant il s'agissait de 50 hommes (91%) et 5 femmes (9%). L’âge moyen était de 26 ans avec des extrêmes de 15 et 70 ans. Le côté gauche était le plus fréquemment atteint (57%). Les étiologies des traumatismes étaient les accidents de la voie publique ( 25 cas), des chutes ( 10 cas), des agressions (10 cas), des traumatismes anodins ( 5 cas), et des accidents du sport ( 5 cas). Trente patients étaient polytraumatisés, avec des lésions associées viscérales (20 cas), et osseuses (10 cas). Sur le plan clinique, l'hématurie macroscopique était présente chez 40 cas. Trois patients se sont présentés en état de choc hémorragique et un patient en état de choc hypovolémique (péritonite urineuse). Les quatre malades ont été opérés en urgence, 3 d'entre eux par néphrectomie totale et le quatrième par lavage péritonéal avec conservation du rein (Grade III sur rein unique). L’échographie rénale a été réalisée chez 49 patients, le traumatisme rénal était diagnostiqué dans 45 cas.

Tous nos patients ont eu une tomodensitométrie rénale. Le bilan radiologique permettait de classé les traumatismes selon l'AAST (American Association for the Surgery of Trauma): en traumatismes mineurs (grade I et II) et majeurs (III, IV, et V). On retrouve ainsi, 14 cas (25%) de lésions grade I, 5cas (9%) de lésions grade II, 21cas (38,2%) de lésions grade III, 13 cas (23,6%) de lésions grade IV, et 2cas (3,6%) de lésions grade V. Concernant notre prise en charge, le traitement conservateur avait concerné 50traumatismes fermés (14 grade I, 5 grade II, 19 grade III, 10 grade IV, et 2 grade V). Cette attitude conservatrice a été efficace pour 33 cas, 14 cas (100%) de lésions grade I, 5 cas (100%) de lésions grade II, 15 cas (80%) de lésions grade III, 9 cas (69%) de lésions grade IV, et aucun cas (0%) de lésions grade V, dont 6 ont bénéficié d’ une montée de sonde JJ devant la persistance de l'extravasation urinaire; les 10 cas restants ont nécessité une chirurgie différée pour hémorragie persistante (diminution de l'Hémoglobine). En effet, le traitement chirurgical a été nécessaire dans 15 cas (8 Grade III, 5 Grade IV, et 2 Grade V), avec une néphrorraphie (en urgence différée), neuf treize néphrectomies totales (4 en urgence immédiate et 9 en urgences différées) dont une était totale et élargie devant un traumatisme sur un adénocarcinome rénal, et chez un seul malade l'attitude était l'abstention, après une laparotomie en urgence devant un tableau de péritonite urineuse suite à un traumatisme Grade III sur un rein unique. Après un recul moyen de 11 mois, La mortalité était nulle; Chez les patients opérés, une fonction rénale et une tension artérielle normales ont été noté avec une nette réparation des lésions lors du bilan radiologique, une urétérohydronéphrose a été perdu de vu dans les suites lointaines. Les patients traités de façon conservatrice ont évolué favorablement.

## Discussion

Les traumatismes de l'ensemble de l'appareil urogénital représente 1% à 5% de la traumatologie; et les traumatismes du rein sont les plus fréquents des lésions traumatiques de cet appareil: 64% [1]. 80 à 90% de ces traumatismes sont dus à des accidents de la voie publique, des chutes et des accidents de sport [2, 3]. L'homme est le plus fréquemment atteint [1]. Le traumatisme rénal peut survenir à tout âge avec une prédominance chez le sujet jeune essentiellement entre 20 et 40 ans [1]. L'hématurie est le maitre symptôme, elle est macroscopique dans 66.6 à 99% [1, 4]. Sa détection doit se faire sur le premier jet d'urine, car celle-ci peut disparaître au cours de la seconde et troisième miction [5]. Les lésions associées sont assez fréquente et peuvent être viscérales, osseuses ou cranio-cérébrales [6]. Le bilan lésionnel initial recommandé actuellement est le scanner spiralé qui doit être réalisé en urgence devant: L'hématurie macroscopique; L'hématurie microscopique(avec choc initial, associée à des lésions nécessitant une évaluation scannographique); Un traumatisme fermé avec décélération majeure, contusion du flanc, ou fracture des côtes flottantes; Le polytraumatisé [7, 8].

La découverte d'une hématurie microscopique chez l'adulte dans le cadre d'un traumatisme fermé sans signes de choc ne justifie pas chez lui, de bilan radiologique systématique [9]. Le scanner permet la classification des traumatismes selon ASST qui est composée de 5 stades [1] (Tableau 1). Les traumatismes rénaux sont majeurs dans seulement 20% des cas et environ 15% des patients présentent des lésions grades III et IV [6]. De nos jours, l'abstention chirurgicale représente une nouvelle approche thérapeutique. Seules l'hémodynamique instable ou les lésions viscérales associées constituent une indication absolue pour une révision chirurgicale en urgence [9]. Les lésions Grades III, et IV font actuellement l'intérêt du traitement conservateur [9, 10]. Ces résultats sont améliorés par l'usage, soit des techniques d'embolisation face à un saignement, soit des techniques de drainage endo-urologique devant les extravasations d'urines prolongées [10]. Au vu des résultats publiés dans la littérature, les auteurs proposent de réaliser une chirurgie réparatrice pour les traumatismes grade 4 avec fragments dé vascularisés s'il existe des lésions intra-abdominales associées, notamment des lésions pancréatiques ou coliques [11]. L'extravasation d'urine isolée est souvent spontanément résolutive; quand elle est majeure ou prolongée, (supérieure à une semaine), la mise en place d'une sonde urétérale peut la diminuer permettant d’éviter un traitement chirurgical qui garde sa place dans les avulsions de la jonction pyélo-urétérale [12]. Quant aux lésions pédiculaires, elles sont rares (1 à 4% de l'ensemble des lésions) et peuvent être surveillées ou faire appel à la chirurgie (néphrectomie totale ou partielle, réparation vasculaire directe, pontage artériel et auto transplantation) [7]. Des études significatives suggèrent qu′une approche non opératoire peut même être appliquée envers des lésions Grade V [12]. Altman et al. [12] ont traité 6 patients Grade V parmi 13 de façon conservatrice, ils ont conclu que la durée du séjour était moindre en soins intensif (4,3 versus 9,0), ainsi l'utilisation des unités de transfusion (2,7 versus 25,2), et les complications. Bien que convaincants, ces données doivent être reconsidérées, car ils sont susceptibles d’être biaisées. La plupart des recommandations publiées suggèrent toujours la néphrectomie de ces reins massivement détruits [13]. Par la suite, une réévaluation radiologique par un uroscanner est justifiée entre le 2ème et le 5ème jour post-traumatique. A long terme, les auteurs préconisent la réalisation d'un scanner à un mois et d'une scintigraphie à 6 mois afin d’évaluer la part de la fonction rénale restante et la présence ou non de cicatrices parenchymateuses.

**Tableau 1:** La classification des traumatismes rénaux fermés selon le Comité Américain de Chirurgie Traumatologique (ASST) [1]

Grade I	Hématome sous-capsulaire sans fracture et sans hématome périrénal.
Grade II	Fracture superficielle (<1 cm) avec hématome péri-rénal.
Grade III	Fracture profonde (>1 cm) sans atteinte de la voie excrétrice.
Grade IV	Fracture profonde avec atteinte de la voie excrétrice et/ou atteinte d'une branche vasculaire principale (artérielle ou veineuse).
Grade V	Rein détruit / atteinte du pédicule rénal / avulsion pyélo-urétérale.

## Conclusion

Par leur fréquence croissante qui est liée à l'augmentation des accidents de la voie publique et de la violence urbaine, les traumatismes du rein demeurent un sujet d'actualité. Ils posent encore des problèmes d'ordre thérapeutique. Etant donné l’évolution favorable chez nos patients ayant eu une simple surveillance et tenant compte des bons résultats de la littérature concernant l'abstention thérapeutique dans le traumatisme rénal fermé majeur, nous pensons qu'en l'absence de lésions intra-abdominales associées ou d'instabilité hémodynamique, la simple surveillance reste la méthode thérapeutique de choix.
